# Structural and Functional Analyses of the Shedding Protease ADAM17 in HoxB8-Immortalized Macrophages and Dendritic-like Cells

**DOI:** 10.4049/jimmunol.1701556

**Published:** 2018-10-24

**Authors:** Anne-Sophie Cabron, Karim El azzouzi, Melanie Boss, Philipp Arnold, Jeanette Schwarz, Marcela Rosas, Jan Philipp Dobert, Egor Pavlenko, Neele Schumacher, Thomas Renné, Philip R. Taylor, Stefan Linder, Stefan Rose-John, Friederike Zunke

**Affiliations:** * Institute of Biochemistry, Christian-Albrechts-University of Kiel, 24118 Kiel, Germany;; † Institute for Medical Microbiology, Virology and Hygiene, University Medical Center Eppendorf, 20246 Hamburg, Germany;; ‡ Institute of Anatomy, Christian-Albrechts-University of Kiel, 24118 Kiel, Germany;; § Division of Infection and Immunity, Systems Immunity Research Institute, Cardiff University School of Medicine, Cardiff CF10 3AT, United Kingdom;; ¶ Department of Molecular Medicine and Surgery, Karolinska Institutet and University Hospital, Solna, SE-171 76 Stockholm, Sweden; and; ‖ Institute of Clinical Chemistry and Laboratory Medicine, University Medical Center Hamburg-Eppendorf, 20246 Hamburg, Germany

## Abstract

A disintegrin and metalloproteinase (ADAM) 17 has been implicated in many shedding processes. Major substrates of ADAM17 are TNF-α, IL-6R, and ligands of the epidermal growth factor receptor. The essential role of the protease is emphasized by the fact that ADAM17 deficiency is lethal in mice. To study ADAM17 function in vivo, we generated viable hypomorphic ADAM17 mice called ADAM17^ex/ex^ mice. Recent studies indicated regulation of proteolytic ADAM17 activity by cellular processes such as cytoplasmic phosphorylation and removal of the prodomain by furin cleavage. Maturation and thus activation of ADAM17 is not fully understood. So far, studies of ADAM17 maturation have been mainly limited to mouse embryonic fibroblasts or transfected cell lines relying on nonphysiologic stimuli such as phorbol esters, thus making interpretation of the results difficult in a physiologic context. In this article, we present a robust cell system to study ADAM17 maturation and function in primary cells of the immune system. To this end, HoxB8 conditionally immortalized macrophage precursor cell lines were derived from bone marrow of wild-type and hypomorphic ADAM17^ex/ex^ mice, which are devoid of measurable ADAM17 activity. ADAM17 mutants were stably expressed in macrophage precursor cells, differentiated to macrophages under different growth factor conditions (M-CSF versus GM-CSF), and analyzed for cellular localization, proteolytic activity, and podosome disassembly. Our study reveals maturation and activity of ADAM17 in a more physiological-immune cell system. We show that this cell system can be further exploited for genetic modifications of ADAM17 and for studying its function in immune cells.

## Introduction

As a member of the a disintegrin and metalloproteinase (ADAM) protease family, ADAM17 performs ectodomain shedding of diverse transmembrane proteins. ADAM17 has been first described as key protease involved in TNF-α shedding (1, 2). Besides TNF-α, its receptors TNFR_I_ and TNFR_II_, the IL-6R, and ligands of the epidermal growth factor receptor have been added to the long list of, to date, more than 80 ADAM17 substrates (3). The important role of ADAM17 in vivo is supported by the fact that deletion of the ADAM17 gene in mice is lethal (4). To study ADAM17 function in vivo, hypomorphic ADAM17^ex/ex^ mice were generated, which are viable and show ∼5% residual ADAM17 expression and no measurable shedding activity (5). Because ADAM17 substrates include membrane-bound cytokines [e.g., TNF-α, cytokine receptors, and the membrane-bound chemokines fractalkine and CXCL16 (6)], ADAM17 turned out to be a key regulator during inflammation. Hence, genetic deletion of ADAM17 or pharmacologic blockade in neutrophils and leukocytes mediated resistance to LPS-induced endotoxemia and protected mice from otherwise lethal septic shock (7, 8). Because ADAM17 processes the IL-6R, ADAM17 plays a decisive role in the IL-6 transsignaling pathway as part of the immune response (9).

Despite the importance of ADAM17 in processing a large range of substrates, the regulatory mechanisms leading to ADAM17 activation and substrate recognition are not fully understood. Transgenic mice overexpressing ADAM17 show no enhancement in substrate cleavage (10), pointing toward strict regulation of protease activity by posttranslational mechanisms. ADAM17 is generated as a proenzyme, and the N-terminal propeptide can act as an autoinhibitor to keep the protease in an inactive state (11, 12). The prodomain of ADAM17 is removed by furin-like convertases at two different sites (13): one located between the propeptide and the metalloprotease domain (referred to as downstream [ds] site) and the second cleavage site found within the prodomain (referred to as upstream [us] site), cleavage of which has been described as a prerequisite to cleavage at the ds site (13).

Another posttranslational modification, which has been claimed to be important for ADAM17 activity, is phosphorylation of the cytoplasmic domain (14–18). Consequently, treatment of cells with phorbol ester (PMA) led to an increase in ADAM17 activity (19, 20). Under physiologic conditions, phosphorylation of ADAM17 is mediated by MAPKs (16) and polo-like kinase 2 (PLK2) at serine 794 (15). However, the importance of phosphorylation of the cytoplasmic tail for ADAM17 activity is under debate because ADAM17-deficient cell lines show normal processing of substrates after reconstitution with ADAM17 variants, in which the entire cytoplasmic region was deleted (21–23). In more recent studies, it was shown that ADAM17 with a short-charged membrane-proximal stretch of 5 aa of the cytoplasmic portion together with a protein tag showed ADAM17-shedding activity (24, 25). Because there is ongoing discrepancy in the field about the role and effect of the various C-terminal (CT) deletion mutants on ADAM17 function, a recent study uses ADAM17 variants devoid of intracellular amino acids, comparing a complete deletion of all 133 cytoplasmic amino acids (ADAM17 ΔCT) with an ADAM17 mutant still exhibiting a residual stretch of six charged amino acids of the cytoplasmic portion (ADAM17 Δ700) (26). Interestingly, the ADAM17 Δ700 variant showed normal activity, whereas the complete deletion of the cytoplasmic tail (ADAM17 ΔCT) resulted in a complete loss of shedding activity (26). This is the reason why in this study we exclusively focus on the function of the ADAM17 Δ700 variant.

Studies addressing the physiologic relevance of posttranslational modifications of ADAM17 have been performed mainly in mouse embryonic fibroblasts (mEF) and in human embryonic kidney (HEK) cells with stimulation induced by the phorbol ester PMA (13, 15, 24, 26, 27). ADAM17 activation by the nonphysiologic stimulator PMA and analysis of mostly cell-extrinsic substrates do not provide a natural environment for studying ADAM17 function. For instance, physiologically relevant stimuli of immune cells include LPS and zymosan, which activate the TLR-4 and TLR-2 as well as the dectin-1 pathway, respectively (28–30). The main goal of the current study was to establish a robust cell model to study ADAM17 regulation and function under more physiologic conditions. Because ADAM17 is a key regulator of the immune system, we chose primary cells of the innate immune system for our experiments. Retroviral transduction of bone marrow–derived macrophages and bone marrow granulocytes from wild-type (wt) and ADAM17^ex/ex^ mice was not efficient enough to allow for studying ADAM17 variants. Hence, we applied an approach using an estrogen-dependent HoxB8 conditional immortalization protocol generating a macrophage precursor cell (MØP) line (31, 32). This cell system allows stable generation of proliferating hematopoietic progenitor cells, which can be further differentiated by estrogen depletion and application of M-CSF and GM-CSF (32). The generated MØP as well as the differentiated mature macrophage-like cells (M-MØ) and dendritic-like cells (GM-MØ) respond to TLR stimulation by NF-κB–induced transcription of proinflammatory genes like TNF-α, IL-1β, and IL-6. Moreover, these cells can be easily transduced by retroviral vectors.

In this study, we applied this cell system to study structural alterations of ADAM17, comparing the results to overexpression studies in mEF and HEK cells. ADAM17^ex/ex^-derived HoxB8 progenitor cells were transduced with cDNAs coding for ADAM17 variants, including ADAM17 Δ700, ADAM17 S794A, and the two furin cleavage–resistant mutants ADAM17 *RVNG* us and *RVNG* ds. ADAM17-reconstituted M-MØ and GM-MØ were examined for expression, cellular localization, and biological activity of the ADAM17 variants. Our data provide evidence for a divergent regulation of ADAM17 protease activity in a physiologic cellular background in which we were able to analyze the role of ADAM17 in the disassembly of podosomes, which might be involved in Ag sampling and recognition (33).

## Materials and Methods

### cDNA constructs and cloning

Expression plasmids of murine ADAM17 mutants (S794A, Δ700, *RVNG* ds, *RVNG* us) were cloned via site-directed mutagenesis PCR using the mADAM17 in the pcDNA3.1 (+) vector template as described (15, 26). All ADAM17 variants were cloned into the retroviral pMXs-IZ vector (34) using the XhoI and NotI restriction site and were myc tagged at the C terminus. All plasmids were verified by DNA sequencing (GATC Biotech).

### Cell culture

MØP were generated from bone marrow of ADAM17^wt/wt^ and ADAM17^ex/ex^ mice as previously described (32, 34). Briefly, CD117-enriched bone marrow cells (using CD117-biotin [BD Biosciences] and anti-biotin-MACS [Miltenyi Biotec]) were infected with an estrogen-dependent Hoxb8 pMXs retroviral vector for conditional immortalization and cultured in RPMI 1640 medium (Thermo Fisher Scientific) containing 10% heat-inactivated FCS (PAA Laboratories), 1% penicillin/streptomycin (Pen/Strep), β-estradiol (1 μM) (Sigma-Aldrich), GM-CSF (10 ng/ml) (ImmunoTools), and puromycin (20 μM/ml) (Roth). After selection in puromycin, the MØP, ADAM17-deficient cells were further transduced with retroviral vectors coding ADAM17 variants (in pMXs-IZ vector) and incubated with zeocin (100 μg/ml) for selection of stably transfected cells. ADAM17-reconstituted MØP were further differentiated to M-MØ and GM-MØ by application of M-CSF and GM-CSF, respectively, in the absence of estrogen (see below and Fig. 2A).

mEF were isolated from E13.5 ADAM17^wt/wt^ and ADAM17^ex/ex^ embryos and immortalized with the SV40 large T Ag as previously described (5). HEK293T cells were purchased from DSMZ (Braunschweig, Germany), and ADAM10- and ADAM17-deficient HEK cells (HEK double knockout [dKO]) were generated via CRISPR/Cas9 system as described (35). mEF and HEK cells were cultured in DMEM containing 10% heat-inactivated FCS and 1% Pen/Strep.

### Transfection and retroviral transduction

HEK cells were transiently transfected with polyethylenimine (PEI) in a 1:3 DNA to PEI ratio and mEF by using X-tremeGENE HP DNA Transfection Reagent (Roche) according to the manufacturer’s instructions. Retroviruses were generated in EcoPacks (Clontech Laboratories) and transfected by using TurboFect (Thermo Scientific). The supernatant of the transfected EcoPacks was concentrated with Retro-X Concentrator (Clontech Laboratories). The concentrated retrovirus was used to infect ADAM17^ex/ex^ MØP with the ADAM17 variants. The cells were incubated with the virus and polybrene (8 μg/ml) for 3 d and selected for stable transduction by zeocin.

### MØP differentiation

For the differentiation in GM-MØ, the MØP were washed three times with RPMI 1640 containing 10% heat-inactivated FCS and 1% Pen/Strep and seeded at a density of 250,000 cells per well in a six-well plate in RPMI 1640 additionally supplemented with 10 ng/ml GM-CSF. Medium was changed every 2 d. On the seventh day of differentiation, the GM-MØ were stimulated with 1 μg/ml LPS (*Escherichia coli* O111:B4; Sigma-Aldrich) or zymosan (25 μg/ml) for 12 h. For M-MØ differentiation, three wash steps with RPMI 1640 containing 10% heat-inactivated FCS and 1× Pen/Strep were applied before seeding at a density of 350,000 cells per well on a six-well plate in RPMI 1640 additionally supplemented with 20 ng/ml M-CSF. The medium was changed every 2 d. The stimulation with LPS (1 μg/ml) or zymosan (25 μg/ml) was done on day 5 of differentiation. Cells and the supernatant were harvested on the next day. Bright light microscopy pictures were taken during the differentiation with a compact inverted CKX41 microscope (Olympus) equipped with a UPLFLN objective (numerical aperture: 0.3).

### ELISA

A total of 250,000 undifferentiated MØP were seeded onto a 12-well plate and stimulated 6 h later with LPS (1 μg/ml) or zymosan (25 μg/ml) overnight. The differentiated MØP were stimulated 12 h before harvesting. mEF cells were seeded at a density of 200,000 cells per well on a six-well plate. After 24 h, cells were transfected as described above. On the next day, mEF were stimulated with PMA (200 nM) for 2 h. In the cell-free supernatant IL-6, TNF-α and soluble TNFR_II_ were measured by ELISA, according to the manufacturer’s instructions. The TNFR_II_ ELISA kit was from R&D Systems. The ELISA kits for IL-6 and TNF-α were from eBioscience.

### Life cell activity assay

A total of 2 × 10^6^ ADAM10- and ADAM17-deficient HEK cells (HEK dKO) were seeded onto a 10-cm dish and transfected the next day with PEI (1:3 DNA/PEI ratio). The following day, cells were detached from cell culture dish by trypsin and seeded onto a 96-well plate (2 × 10^5^ cells per well). The next day, the medium was replaced by phenolred-free medium, and quenched fluorogenic peptide (Abz-LAQAVRSSSR-Dpa; TACE-substrate IV [no. 616407; Calbiochem]) was added to a concentration of 20 μM. Cell surface activity of ADAM17 was measured in a plate reader (Tecan Infinite 200 Pro) for 6000 s at emission 405 nm and extinction 320 nm. ADAM17 cell surface activity was determined by calculating the area under curve of the fluorescent signal over time.

### Flow cytometry

MØP suspension cells were counted, 250,000 cells were collected, and the differentiated MØP were mechanically detached. All cells were washed three times with ice-cold PBS and blocked for 10 min in PBS containing 1% BSA and 1% Fc block (blocking buffer [BioLegend]) and incubated with the fluorescence-coupled Abs diluted 1:100 in blocking buffer for 1 h at 4°C. Afterwards, the cell suspension was washed two times with ice-cold PBS containing 1% BSA. The staining was fixed with 10% lysis/fixation solution (BioLegend) in distilled water for 15 min at room temperature. Before performing flow cytometry analysis (FACS Canto II; BD Bioscience), the cells were washed again two times with ice-cold PBS containing 1% BSA. A total of 30,000 events were gated using the forward scatter area (FSC-A) and forward scatter height (FSC-H) and only CD45^+^ cells were used for further analyses. All used markers were compensated before the flow cytometry analysis. All data analysis was performed using the flow cytometry analysis software FlowJo (Tree Star).

The following Abs were used: brilliant violet 510 anti-mouse CD45 (clone 30-F11; Biolegend), brilliant violet 421 anti-mouse CD115 (clone AFS98; Biolegend), PE anti-mouse CD11b (clone M1/70; BD Biosciences), APC anti-mouse F4/80 (clone BM8; Biolegend), FITC anti-mouse CD11c (clone N418; Biolegend), PE/Cy7 anti-mouse CD206 (clone C068C2; Biolegend), and APC/Fire anti-mouse MHCII (clone M5/114.15.2; Biolegend).

For ADAM17 cell surface staining, HEK cells were transfected as described above, harvested, and blocked in 1% BSA in PBS for 10 min at 4°C. The MØP were harvested via centrifugation (1000 × *g*, 5 min, 4°C), washed with PBS, and blocked (1% BSA and 1% Fc block, 10 min, 4°C). Afterwards, the cell suspension was stained with the primary Ab (1:100 diluted in 1% BSA in PBS) for 1 h at 4°C, washed with 1% BSA in PBS, and incubated with the secondary Ab (1:100 in 1% BSA in PBS) for 1 h at 4°C. Before flow cytometry analysis, the cells were washed and resuspended in 1% BSA and PBS. To stain dead cells, the MØP suspension was incubated for 5 min with 25 ng of 7-AAD (no. 420403; Biolegend) before flow cytometry analysis.

The following Abs were used: primary Ab anti-ADAM17 10.1 (polyclonal Ab against the extracellular portion of murine ADAM17) and secondary Ab donkey anti-rabbit IgG Alexa Fluor 488 (no. A21206; Thermo Fisher).

### Immunofluorescence analysis

A total of 250,000 MØP per well were seeded in a six-well plate on glass cover slips. After differentiation, cells were stimulated with LPS (1 μg/ml) or zymosan (25 μg/ml) for 12 h. HEK cells were seeded at a density of 200,000 cells per well on a six-well plate and transfected as described above on the following day. Twenty-four hours after transfection, the cells were stimulated with PMA (200 nM) for 2 h. Cells were washed with PBS, fixed with 4% PFA in PBS for 15 min at room temperature, and permeabilized with 0.3% Triton X-100 (Sigma-Aldrich) in PBS for 30 min. Cells were blocked in blocking buffer (2% BSA, 5% heat-inactivated FCS, and 0.3% Triton in PBS) for 60 min. The primary Abs were diluted in blocking buffer and incubated at 4°C overnight. Cells were washed three times with PBS containing 0.3% Triton X-100 and incubated with secondary Abs diluted 1:500 in blocking buffer. Cells were washed three times with 0.3% Triton X-100 in PBS, one time with PBS, and stained with DAPI as part of the mounting mix consisting of DABCO (Sigma-Aldrich) and MOWIOL (Merck Millipore). Stained cells were viewed and photographed with an FV1000 confocal laser scanning microscope (Olympus) equipped with a U Plan S Apo 100× oil immersion objective (numerical aperture: 1.40). Digital images were processed using FV10-ASW 4.2 Viewer (Olympus). The Pearson index was used to express colocalization of two stainings, which was calculated by an integrated tool within the FV10-ASW software. Transfected HEK cells were marked by a region of interest, and colocalization of two stainings was determined by the software, resulting in values between 0 and 1. The value 1 indicates a 100% colocalization of both markers (36). Primary Abs used are as follows: anti-PDIA6 (1:750, ab11432; Abcam), anti-myc (1:250, clone 9B11 no. 2276; Cell Signal), KDEL (1:100, clone 10C3; Enzo), anti-human ADAM17 [1:50, A300D, in-house production (37)], anti-ADAM17 10.1 (1:100), and anti-ADAM17 18.2 (1:100) (polyclonal Abs generated by Pineda Antikörper-Service, Berlin, Germany). Secondary Abs were purchased from Thermo Scientific: goat-anti-mouse Alexa Fluor 488, goat-anti-rabbit Alexa Fluor 488, goat-anti-mouse Alexa Fluor 594, and goat-anti-rabbit Alexa Fluor 594. For staining of ADAM17 in M-MØ and GM-MØ, the anti-myc Ab was used because stably transfected ADAM17 constructs were myc tagged at the C terminus.

### Western blot analysis

Differentiated MØP, mEF, and HEK cells were mechanically detached from the culture dish. MØP suspension cells were pelleted by centrifugation (1000 × *g*, 5 min, 4°C). All cells were washed with ice-cold PBS and lysed in lysis buffer containing 10 mM 1.10-phenanthroline (Merck Millipore). The MØP lysis buffer contained 1% Triton X-100, 150 mM NaCl, 50 mM Tris-HCL (pH 7.4), and two tablets of complete protease mixture inhibitor (Roche) in 50 ml. The lysis buffer used for the mEF and HEK cells additionally contained 1% IGEPAL (Nonidet P-40) and 2 mM EDTA. The amount of total protein was determined by BCA assay (Thermo Scientific). Aliquots of the lysates were supplemented with 5× SDS sample buffer (0.3 M Tris-HCl, pH 6.8, 10% SDS, 50% glycerol, 20% mercaptoethanol, 5% bromophenol blue) and heat inactivated at 95°C for 5 min. Equal amounts (30–40 μg total protein) were separated by SDS-PAGE and transferred to a PVDF membrane (Merck Millipore). The following Abs were used for detection: anti-ADAM17 10.1 (polyclonal Ab against the extracellular portion of murine ADAM17 [peptide: CEVKPGRHFNMAKSFPNEEK]), anti-ADAM17 18.2 (polyclonal Ab against the intracellular portion of murine ADAM17 [peptide: RLQALQPAAMMPPVSAAPKL; both Pineda Antikörper-Service]), anti-ADAM17 (polyclonal Ab against intracellular domain of ADAM17, ab39162; Abcam), anti-human ADAM17 [A300D, in-house production (37)], anti-iRhom2 (RHBDF2) (ab116139; Abcam), anti-TNFR_II_ (ab221921; Abcam), anti-pro–TNF-α [in-house production, kindly provided by B. Schröder, Technische Universität Dresden, Dresden, Germany (26)], anti-β-actin (A5441; Sigma-Aldrich), and anti-ADAM10 (ab124695; Abcam). As secondary Abs, goat anti-rabbit HRP and goat anti-mouse HRP (both Dianova) were used. For protein enrichment by Con A (Sigma-Aldrich) precipitation, 1000 μl of cell lysate was incubated with 30-μl Con A beads overnight at 4°C under constant agitation. Samples were spun down for 15 min at 4000 × *g* and washed three times with PBS. The pellet was incubated with 100 μl of PBS and 100 μl of 5 × loading buffer (see above), heated to 95°C for 10 min, and analyzed on SDS-PAGE.

### Immunoprecipitation and furin assay

Cells were lysed, and protein was quantified as described above. Afterwards, 1 mg of total protein was immunoprecipitated with 3 μg of anti-ADAM17 Ab (no. ab39162; Abcam) overnight at 4°C. The used Protein G Dynabeads (no. 10004D; Thermo Fisher Scientific) were blocked with 1% BSA in PBS overnight at 4°C. The blocked beads were added to the lysates and incubated for 1 h at 4°C. After three washing steps with lysis buffer, 1 × SDS sample buffer was added to the beads and heated to 65°C for 15 min. For the furin cleavage assay, the beads were incubated in furin assay buffer (100 mM HEPES, 1 mM CaCl, 1 mM 2-ME, 0.5% Triton X-100) with 1 U recombinant furin (no. P8077S; New England Biolabs) for 30 min at 30°C. Afterwards, 5 × SDS sample buffer was added and boiled. The immunoprecipitated proteins were further analyzed by Western blotting.

### Electron microscopy

For scanning electron microscopy (SEM), cells were seeded on glass slides and differentiated to the desired cell type. Subsequently, cells were fixed in 3% glutaraldehyde in PBS for 30 min prior to three times washing in PBS. Prior to ascending ethanol steps (30, 40, 50, 70, 96, 100%), cells were fixed in 2% osmium tetroxide for 30 min. After critical point drying and sputtering with gold, samples were imaged on a Philips XL20 SEM.

### Analysis of podosomes

MØP were differentiated by M-CSF for 6 d and stimulated with LPS for 24 h as described above. Subsequently, cells were fixed with 3.7% formaldehyde, permeabilized with 0.5% Triton X-100 and PBS (pH 7.5) for 10 min and stained for podosome markers F-actin (Alexa-Fluor-488– or Alexa-Fluor-568–phalloidin; Molecular Probes, Eugene, OR) and vinculin (mAb V9264, 1:500; Sigma-Aldrich) to highlight podosome rings. CellMask Deep Red plasma membrane stain (no. C10046; Thermo Fisher Scientific) was used to stain the cytoplasm and nuclei at a concentration of 2 μg/ml. Images of fixed samples were acquired with a confocal laser-scanning microscope (DMI 6000 with a TCS SP5 AOBS confocal point scanner; Leica) equipped with an oil-immersion HCX PL APO 63× numerical aperture 1.4–0.6 objective. Podosome number and cell area analysis were performed using an ImageJ (National Institutes of Health, Bethesda, MD) macro (38). The analysis was performed with three different donors with 30 cells for each condition (*n* = 3 × 30).

### Protein modeling

Protein modeling was performed with Swiss Model Expasy (http://swissmodel.expasy.org/) and the Chimera package (University of California; www.cgl.ucsf.edu/chimera).

### Data analysis and statistics

All values are expressed as the mean ± SEM. For data analysis, Excel (Microsoft) and GraphPad Prism version 6.00 for Mac (GraphPad Software) were used. Differences among mean values were analyzed as indicated in the figure legends by two-tailed, unpaired Student *t* test or one-way ANOVA, followed by a Tukey multiple comparison test using GraphPad Prism6 software for multiple samples, when applicable. In all analyses, the null hypothesis was rejected at *p* < 0.05, with *p < 0.05, **p < 0.01, ***p < 0.005, and ****p < 0.001.

## Results

### Functional and structural characterization of ADAM17 mutants in mEF and HEK cells

**FIGURE 1.**
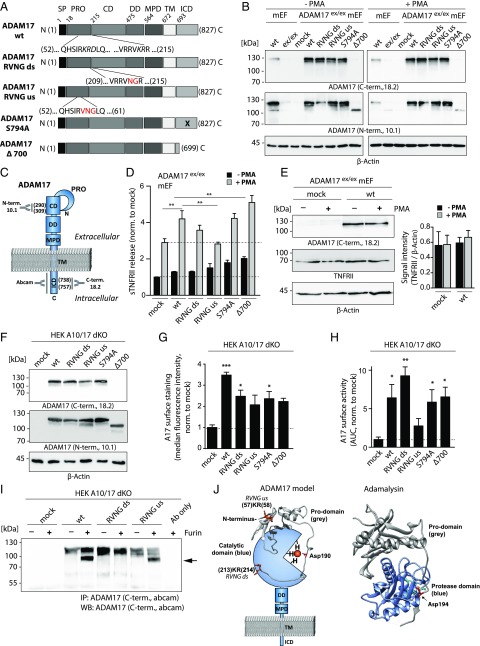
Functional and structural analyses of ADAM17 mutants in mEF and HEK cells. (**A**) Schematic overview of the 827-aa-long murine ADAM17 protein subdivided in different domains and ADAM17 mutants analyzed in this study. Mutations introduced in the prodomain to generate the *RVNG* ds and *RVNG* us mutants are indicated by red letters. The location of the S794A point mutation is indicated by a black cross, and the ADAM17 Δ700 mutant is truncated after aa 699. (**B**) Immunoblot of endogenous ADAM17 in wt mEF and transiently overexpressed ADAM17 in mEF exhibiting an ADAM17 knockdown (ADAM17^ex/ex^). Cells were nonstimulated (−PMA) or stimulated with PMA for 2 h. C-terminal (18.2) as well as N-terminal (10.1) Abs were used to detect ADAM17, indicating uniform expression of all ADAM17 mutants. The Δ700 can only be detected by the N-terminal Ab. β-Actin was used as loading control. (**C**) Schematic model of ADAM17 protein indicating binding sites of used Abs. The polyclonal N-terminal ADAM17 Ab 10.1 was designed against a peptide (aa 290–309) in the catalytic domain (CD). Both C-terminal Abs (ab39162 and house-made 18.2; Abcam) are polyclonal and were designed against a cytoplasmic epitope. The 18.2 Ab was designed to bind a peptide (aa 738–757) within the intracellular domain (ICD). (**D**) ELISA of endogenous TNFR_II_ in supernatants of ADAM17^ex/ex^ mEF reconstituted with each respective ADAM17 mutant, showing constitutive shedding activity (black bars) and TNFR_II_ shedding after a 2-h PMA stimulation (gray bars). The dotted line marks baseline TNFR_II_ shedding (*n* = 3 from three individual transfections). (**E**) Endogenous TNFR_II_ protein level in ADAM17^ex/ex^ mEF after mock transfection and reconstitution with ADAM17 wt with and without a 2-h PMA stimulation. Signal intensity analysis of TNFR_II_ normalized to loading control (β-actin) indicates unchanged TNFR_II_ level. (**F**) Representative immunoblot of ADAM17 mutants transiently overexpressed in ADAM10/ADAM17 double-deficient HEK cells (HEK A10/A17 dKO) detected by a C-terminal (18.2) as well as N-terminal Ab (10.1) showing equal ADAM17 protein level. β-Actin was used as loading control. (**G**) Cell surface FACS analysis of ADAM17 overexpressed in HEK A10/A17 dKO cells stained with extracellular N-terminal Ab 10.1. Single cells (FSC-A × FSC-H) were gated and plotted for secondary Ab signal (Alexa Fluor 488). Bar graph shows median fluorescence intensity after normalization to mock transfection (*n* = 3 from three individual transfections). (**H**) ADAM17 life cell surface activity assay in ADAM17-reconstituted HEK A10/17 dKO cells by fluorogenic peptide cleavage assay. Protease activity was determined by measuring fluorescence intensity of the cleaved peptide on living cells for 6000 s. The area under the curve of fluorescent signal over time was calculated and normalized to the mock-transfected control (*n* = 3 from three individual transfections). (**I**) Representative Western blot of three individual experiments (anti C-terminal Ab [Abcam]) of a furin cleavage assay. Lysates of ADAM17-transfected HEK A10/A17 dKO cells were immunoprecipitated by a C-terminal Ab (Abcam) and incubated with 1 U of recombinant furin. Upper ADAM17 band (∼120 kDa) indicates full-length and nonfurin-processed ADAM17 form. After furin cleavage of the prodomain, the mature ADAM17 migrates at ∼90 kDa (black arrow). (**J**) Protein model of ADAM17 highlighting catalytic and prodomain (gray). In orange, the us and ds furin cleavage sites are indicated. The prodomain of ADAM17 and its orientation toward the catalytic domain were modeled on the basis of the previously published adamalysin crystal structure [right; PDB:3P24 (39)] using Swiss Model Expasy. The red highlighted Asp194 in adamalysin is analog to the Asp190 in the prodomain of ADAM17. Both aspartates interact with a zinc ion (orange dot). Molecular imaging was performed using UCSF Chimera. For statistical analyses, a two-sided Student *t* test was used in (E), exhibiting no statistically significant differences. A one-way ANOVA followed by a Tukey multiple comparison test was applied in (D), (G), and (H). CD, catalytic domain; DD, disintegrin domain; ICD, intracellular domain; MPD, membrane proximal domain; PRO, prodomain; SP, signal peptide; TM, transmembrane domain. *p < 0.05, **p < 0.01, ***p < 0.005.

We generated four ADAM17 mutants by introducing point mutations and a C-terminal truncation after residue 699, leaving a cytoplasmic rest of 6 aa (ADAM17 Δ700). Two furin-resistant mutants were generated by introduction of an *RVNG* motif in either the ds (*RVKR*) or us (*RKRD*) furin cleavage site. Furthermore, we mutated the phosphorylation site of ADAM17 by replacing Ser^794^ with Ala (Fig. 1A). To study posttranslational processes of ADAM17, mutants were analyzed for expression, cellular localization, and proteolytic activity. Initial experiments were performed in mEF and HEK cells on an ADAM17-deficient background. mEF were obtained from hypomorphic ADAM17^ex/ex^ animals and transiently transfected with the respective ADAM17 mutants. Equal protein expression of all ADAM17 variants was verified by SDS-PAGE and Western blotting using C- and N-terminal ADAM17 Abs under unstimulated (−PMA) and stimulated (+PMA) conditions (Fig. 1B). It should be noted that the cytoplasmic deletion mutant (Δ700) can only be detected by the N-terminal ADAM17 Ab (Fig. 1B). Epitopes of used Abs are visualized on a schematic ADAM17 protein (Fig. 1C). For functional analysis, ADAM17-transfected mEF were stimulated by PMA. Cell supernatants were analyzed for endogenous TNFR_II_ shedding by ELISA, showing an increase in soluble TNFR_II_ levels, indicating enhanced ADAM17 activity in ADAM17-reconstituted mEF in comparison to mock-transfected cells (Fig. 1D). Mutants ADAM17 *RVNG* ds, S794A, and Δ700 exhibited higher TNFR_II_-shedding activities than mock transfectants, and only the *RVNG* us mutant showed no protease activity after PMA stimulation (Fig. 1D). No changes in TNFR_II_ protein expression were observed under the experimental conditions used (Fig. 1E, Supplemental Fig. 1A).

We further used ADAM10/ADAM17 dKO HEK cells for structural and functional studies of ADAM17 variants, in which absence of ADAM10 and ADAM17 was verified by Western blotting and immunofluorescence (Supplemental Fig. 1B, 1C). ADAM17 expression in reconstituted HEK A10/A17 dKO cells was verified by Western blotting (Fig. 1F, Supplemental Fig. 1D) and immunofluorescence (Supplemental Fig. 1G). For validation of ADAM17 bands on Western blots, three different Abs were used: two C-terminal (18.2; Abcam) and one N-terminal (10.1; Fig. 1F, Supplemental Fig. 1D; see also Fig. 1C). The mature form of overexpressed ADAM17 protein (∼90 kDa) could only be detected by Western blotting after enrichment of the protein by immunoprecipitation with a C-terminal ADAM17 Ab (Supplemental Fig. 1E). Further, no differences in glycosylation patterns between the different ADAM17 variants could be found as indicated by a peptide-*N*-glycosidase F digest (Supplemental Fig. 1F).

Cell surface localization of all ADAM17 variants in HEK cells was verified by FACS analysis, using an N-terminal Ab (Fig. 1G). To further determine enzyme activity on the cell surface, a fluorogenic peptide cleavage assay was performed, indicating activity for all ADAM17 variants comparable to the wt except for the *RVNG* us mutant (Fig. 1H). To further analyze the intracellular localization of ADAM17 mutants before and after PMA stimulation in HEK A10/A17 dKO cells, costainings with an endoplasmic reticulum [ER] marker were performed (Supplemental Fig. 1G). Interestingly, highest ER colocalization was found for the ADAM17 Δ700 variant under nonstimulated and stimulated conditions (Supplemental Fig. 1G, 1H). This could indicate less efficient trafficking of this ADAM17 mutant from the ER to other cellular compartments, such as the cell surface, compared with the ADAM17 wt (Supplemental Fig. 1G, 1H; see also Fig. 1G). However, cell surface localization of the proteolytically active ADAM17 variants seems to be sufficient for protease activity as indicated by the TNFR_II_ ELISA in mEF (Fig. 1D) and the fluorogenic peptide cleavage assay in HEK cells (Fig. 1H). To confirm the described furin resistance of the ADAM17 mutants (*RVNG* ds and *RVNG* us), their maturation was analyzed by a recombinant furin assay and Western blotting. Immunoprecipitated ADAM17 from transfected HEK A10/A17 dKO cells was incubated with recombinant furin, resulting in the immature form of ADAM17 (∼120 kDa) and, in case of cleavage of the prodomain, a mature ∼90 kDa–sized form (Fig. 1I). Maturation of ADAM17 protein could be observed for ADAM17 wt and surprisingly also, but to a lesser extent, the *RVNG* us mutant but not for the *RVNG* ds mutant (Fig. 1I). Modeling the prodomain of ADAM17 on the basis of the adamalysin crystal structure [previously published structure PDB:3P24 in Fig. 1J, right (39)] shows the potential orientation and interaction of the prodomain with the catalytic domain (Fig. 1J). Furin cleavage sites (us: aa 57–58; ds: aa 213–214) are shown in orange (Fig. 1J), illustrating how the cleavage of the prodomain and, thus, maturation of ADAM17 could be mediated.

### The macrophage progenitor system (MØP) on ADAM17^ex/ex^ background

**FIGURE 2.**
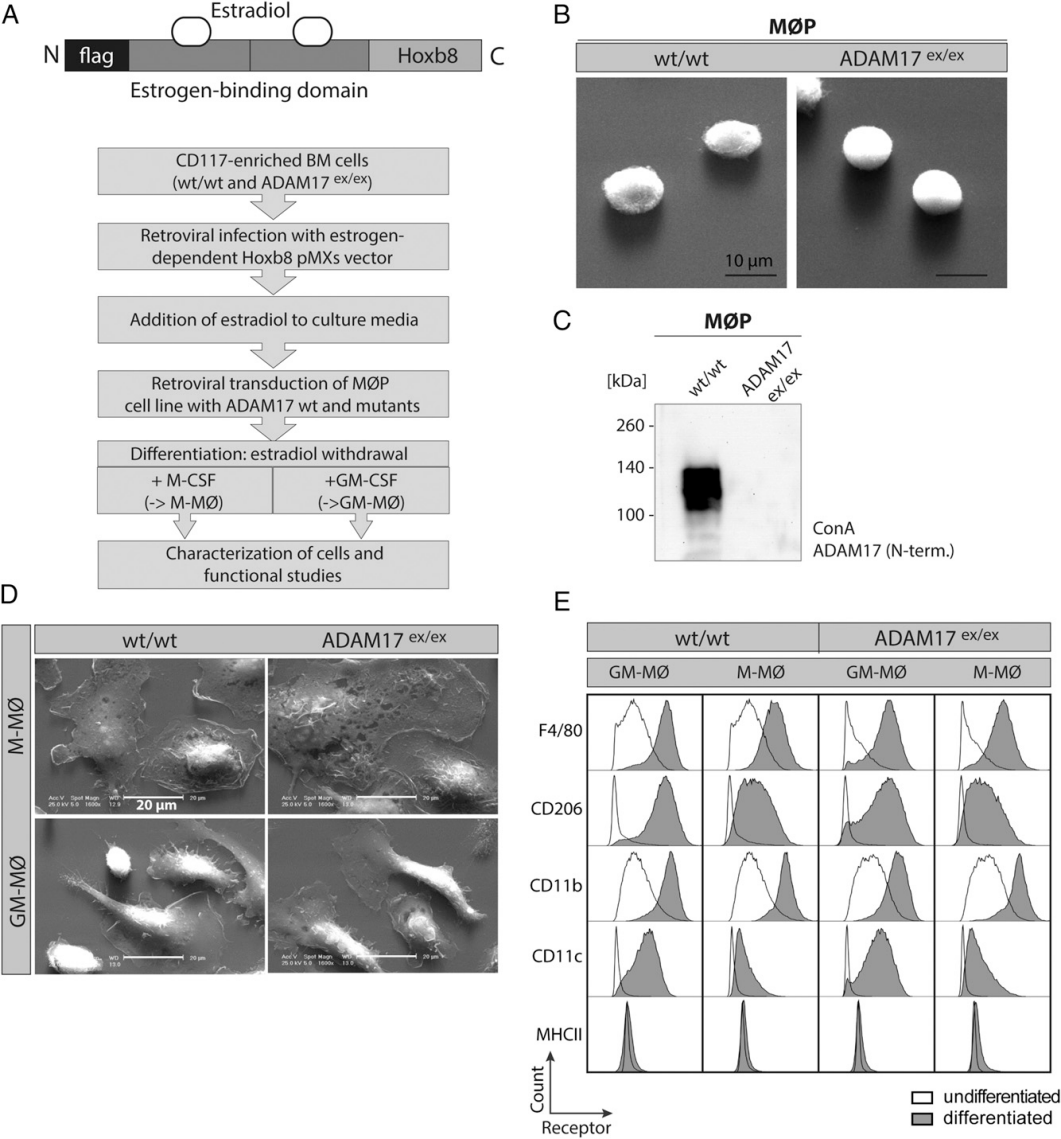
Establishment and characterization of MØP on wt and ADAM17^ex/ex^ background. (**A**) Flow diagram of protocol to establish MØP from wt and ADAM17^ex/ex^ mice and further differentiation to M-MØ and GM-MØ by application of M-CSF and GM-CSF. Top, Scheme of the retroviral HoxB8 expression plasmid, which is used for estrogen-dependent immortalization of CD117-positive bone marrow–derived cells. (**B**) Electron microscopic picture of HoxB8-immortalized macrophage progenitor cells gained from wt and ADAM17^ex/ex^ mice, indicating equal shape and size of MØP. Scale bar, 10 μm. (**C**) Representative immunoblot of Con A–enriched ADAM17, validating protein expression in wt but not in ADAM17^ex/ex^ MØP using an N-terminal (10.1) Ab. (**D**) EM was performed on differentiated M-MØ and GM-MØ from wt and ADAM17^ex/ex^ MØP. M-MØ from either genetic background exhibit characteristic round cell structure, whereas the GM-MØ show a narrower cell shape with extensions. Scale bar, 20 μm. (**E**) FACS analysis of cell surface markers of differentiated M-MØ and GM-MØ in wt and ADAM17^ex/ex^ background in comparison with undifferentiated MØP. Shaded histograms represent the receptor staining of differentiated M-MØ or GM-MØ. Bold lines indicate receptor signal of undifferentiated MØP. Signal of the receptor staining increases according to differentiation and is independent of ADAM17 presence. The histograms are representative of plots from three independent experiments for each marker.

Because there still is debate and controversy on posttranslational regulation processes of ADAM17, we analyzed ADAM17 maturation and activity under more physiologic conditions. We exploited a cell system of conditionally HoxB8-immortalized macrophagic progenitor cells (MØP) (32) to study ADAM17 cellular biology. From the bone marrow of wt and ADAM17^ex/ex^ mice, hematopoietic stem cells were enriched by selection for CD117 and infected with an estrogen-regulated HoxB8 expression vector, allowing proliferation of macrophage progenitors (MØP) in the presence of estrogen (32) (Fig. 2A). The progenitor cells can easily be transduced by retroviral vectors coding for the different variants of ADAM17 shown in Fig. 1A. After selection of stably transduced MØP, these were differentiated into either macrophage- or dendritic-like cells by application of M-CSF (M-MØ) or GM-CSF (GM-MØ), respectively (Fig. 2A). First, immortalized MØP as well as differentiated M-MØ and GM-MØ on wt or ADAM17^ex/ex^ background were characterized by imaging, Western blotting, and FACS analysis (Fig. 2B–E). Electron microscopy (EM) pictures verified characteristic morphology of undifferentiated macrophagic precursor cells, exhibiting similar size and appearance of wt and ADAM17^ex/ex^-derived MØP (Fig. 2B). Furthermore, Con A precipitation and subsequent immunoblotting confirmed ADAM17 expression in wt but not in ADAM17^ex/ex^-derived MØP (Fig. 2C). After differentiation of the precursor cells to mature M-MØ and GM-MØ, EM pictures revealed cell type–specific morphology of M-MØ and GM-MØ, with no differences between wt and ADAM17^ex/ex^-derived cells (Fig. 2D). Further, M-MØ and GM-MØ showed increased expression of F4/80, CD206, CD11b, and CD11c in comparison with undifferentiated MØP (Fig. 2E). As expected, GM-CSF differentiated cells exhibited higher expression of CD206 and CD11c in comparison with the M-CSF differentiated cells (Fig. 2E) (32). Interestingly, in contrast to mEF cells, in M-MØ, ADAM17-mediated shedding of TNF-α and TNFR_II_ could be stimulated with the immunologically relevant stimuli LPS or zymosan, indicating the usefulness of our cell system to study ADAM17 activation under physiologic conditions (Supplemental Fig. 2A, 2B).

### Using genetic modification of MØP to study ADAM17 biology

**FIGURE 3.**
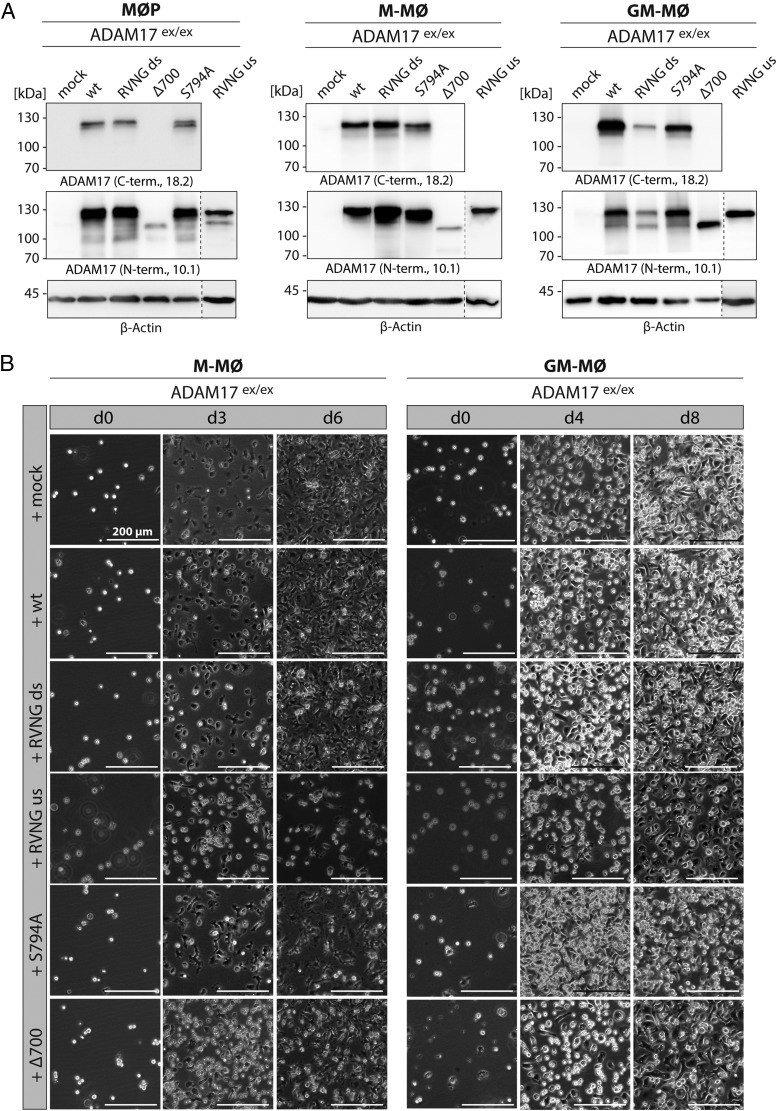
Using the MØP system to study ADAM17 function. (**A**) MØP with ADAM17-deficient background (ADAM17^ex/ex^) were stably reconstituted with ADAM17 variants and further differentiated to M-MØ and GM-MØ. By immunoblotting, stable protein expression of each ADAM17 variant was regularly verified using C-terminal (18.2) as well as N-terminal (10.1) Abs. The presented immunoblots are representative for at least four independent differentiations. β-Actin was used as loading control. (**B**) To ensure equal and complete differentiation of MØP to M-MØ and GM-MØ, cell morphology was monitored by light microscopy for each ADAM17-reconstituted cell line. M-CSF was applied for complete differentiation to M-MØ until day 6 (d6), and representative pictures are shown for day 3 and day 6. Application of GM-CSF had to be continued until day 8 to ensure complete differentiation to GM-MØ, and representative pictures were taken at day 4 and day 8. Undifferentiated MØP are suspension cells, becoming adherent during differentiation growing in characteristic monolayers. Scale bar, 200 μm.

To study ADAM17 function in the precursor as well as in the differentiated macrophage- and dendritic-like cells, MØP were transduced with C-terminally myc-tagged ADAM17 variants described above (Fig. 1A). Stable and equal expression of each ADAM17 construct in the different cell lines (MØP, M-MØ, and GM-MØ) was verified by immunoblotting with two different C-terminal, an anti-myc, and an N-terminal ADAM17 Ab (Fig. 3A, Supplemental Fig. 2C). Furthermore, the ability to differentiate in M-MØ or GM-MØ after transduction with an ADAM17-encoding cDNA was confirmed by light microscopy and FACS analysis (Fig. 3B, Supplemental Fig. 2D). M-MØ differentiation was monitored up to day 6, and GM-MØ differentiation was monitored until day 8. Both time points were chosen to ensure complete differentiation in either cell type (Fig. 3B). Undifferentiated MØP (day 0) were suspension cells, which became adherent during differentiation into macrophages or dendritic-like cells and grew in monolayers (Fig. 3B). The rate of differentiation for every ADAM17-reconstituted MØP line into M-MØ and GM-MØ was evaluated by cell morphology analyzing shape and size using light microscopy. Differentiation did not differ between ADAM17 mutants, indicating that this process is ADAM17 independent, and ADAM17 mutants do not influence macrophage development (Fig. 3B). This result was supported by FACS analyses showing an increase in the immune cell surface markers F4/80, CD206, CD11b, and CD11c in ADAM17-reconstituted M-MØ and GM-MØ as compared with the precursor cells on day 6 for M-MØ and day 8 for GM-MØ (Supplemental Fig. 2D).

### Analyzing function and cellular localization of ADAM17 variants in the MØP system

**FIGURE 4.**
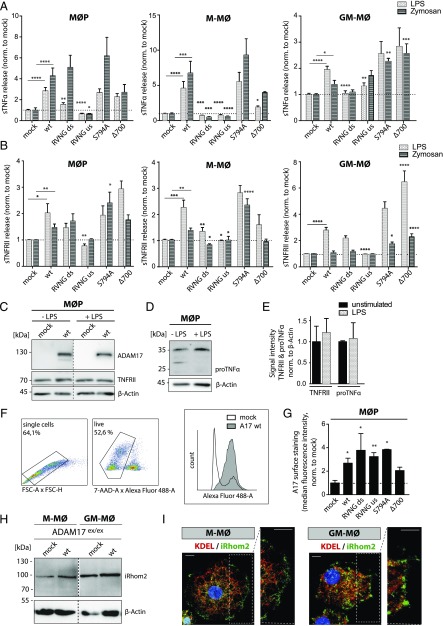
Analyzing function and cellular localization of ADAM17 mutants in primary immune cells. TNF-α– (**A**) and TNFR_II_– (**B**) shedding ELISA of cell supernatant of stably ADAM17-reconstituted ADAM17^ex/ex^ MØP, M-MØ, and GM-MØ after stimulation with LPS (light gray) and zymosan (dark gray). Results are shown normalized to the mock-transfected control, and dotted lines indicate baseline shedding activity (*n* = 9–12, derived from three to four independent rounds of differentiation). Representative immunoblot of the ADAM17 substrates TNFR_II_ (**C**) and pro–TNF-α (**D**) in MØP lysates without and after LPS stimulation. For detection of substrates, Abs targeted against cytoplasmic epitopes were used. (**E**) Densitometric analysis of the ∼70-kDa-sized band of TNFR_II_ as well as the main form of pro–TNF-α (∼35 kDa) normalized to loading control β-actin indicate no differences of substrate level after LPS stimulation compared with unstimulated conditions (*n* = 3). (**F** and **G**) Cell surface staining of ADAM17 in stably ADAM17-transfected ADAM17^ex/ex^ MØP by FACS analysis using an N-terminal ADAM17 Ab (10.1). (F) Gating strategy for analysis: single cells were gated (FSC-A × FSC-H), then live cells (7-AAD negative) were separated and plotted for secondary Ab signal (Alexa Fluor 488). (G) Median fluorescence intensity normalized to mock-transfected cells. Dotted line indicates baseline fluorescence (*n* = 3). (**H**) Immunoblot of iRhom2 in M-MØ and GM-MØ in ADAM17-deficient background and after reconstitution with ADAM17 wt. β-Actin was used as loading control. (**I**) Representative immunofluorescence staining of iRhom2 and KDEL as ER marker in M-MØ and GM-MØ. The white box shows magnification of cell surface structures. Scale bar, 10 μm. If not indicated differently, statistical significance is shown in comparison with the respective ADAM17 wt (A and B) or mock control (G), using a one-way ANOVA, followed by a Tukey multiple comparison test in (A), (B), and (G). A two-sided Student *t* test was applied in (E), exhibiting no significant differences. *p < 0.05, **p < 0.01, ***p < 0.005, ****p < 0.001.

MØP, M-MØ, and GM-MØ expressing different ADAM17 variants were analyzed for shedding of the endogenous ADAM17 substrates TNF-α and TNFR_II_ by ELISA after stimulation with LPS and zymosan (Fig. 4A, 4B). To validate the assay and to ensure efficient and identical activation of ADAM17-reconstituted cell lines by LPS and zymosan, inflammatory protein-like IL-6 as well as the chemokine (C-C motif) ligand 4 were measured, showing similar ADAM17-independent cell stimulation (Supplemental Fig. 3A, 3B). Only the ADAM17 S794A variant exhibited increased IL6 levels after stimulation in M-MØ (Supplemental Fig. 3A, middle panel). To confirm that soluble TNF-α and soluble TNFR_II_ release resulted from ADAM17 activity, a specific ADAM10 inhibitor (GI254023×) and a combined ADAM10 and ADAM17 inhibitor (GW280264×) (40, 41) were added to the MØP prior to stimulation. As shown in Supplemental Fig. 3C, the ADAM10 inhibitor GI did not significantly reduce shed TNF-α levels, whereas the ADAM10/ADAM17 inhibitor GW decreased TNF-α shedding when ADAM17 was present. Further, it was verified that substrate levels (TNFR_II_ and TNF-α) were neither influenced by LPS stimulation nor stable ADAM17 expression in the MØP system (Fig. 4C–E).

Constitutive shedding of TNF-α in all three monocytic cell lines was negligible for all ADAM17 variants in comparison with the mock-transfected control, with no ADAM17 mutant being significantly different from wt ADAM17 (Supplemental Fig. 3D). Interestingly, shedding of TNFR_II_ was significantly increased for the Δ700 and S794A mutants in GM-MØ under unstimulated conditions (Supplemental Fig. 3E, right panel). After stimulation of MØP, M-MØ, and GM-MØ with either LPS or zymosan, efficient TNF-α shedding was observed in cells expressing ADAM17 wt, S794A mutant, and Δ700 mutant (Fig. 4A). For the two *RVNG* mutants (ds and us), diminished TNF-α cleavage was found in M-MØ and GM-MØ (Fig. 4A), underlining the importance of the furin-mediated removal of the propeptide on the ds as well as on the us furin cleavage site for ADAM17 activation (13). Similar results for both *RVNG* mutants (ds and us) were obtained for a second ADAM17 substrate, TNFR_II_ (Fig. 4B). Notably, TNFR_II_ shedding was less efficiently activated by zymosan in all three cell types independent of reconstituted ADAM17 variants (Fig. 4B). Similar to TNF-α shedding (Fig. 4A), the ADAM17 Δ700 mutant and the PLK2 phosphorylation-resistant S794A mutant processed TNFR_II_ efficiently, confirming that the C-terminal part and the PLK2 phosphorylation site are dispensable for protease activity in the analyzed immune cells (Fig. 4B). To further study maturation and the role of cellular localization of ADAM17, intracellular immunofluorescence studies were performed by costaining of ADAM17 and ER marker protein disulfide-isomerase in differentiated M-MØ and GM-MØ after LPS stimulation (Supplemental Fig. 4). Immunofluorescent staining of M-MØ (top panel) and GM-MØ (bottom panel) cells showed characteristic cell morphology. To some extent, all ADAM17 variants (red) showed an overlap with the ER marker protein disulfide-isomerase (green) as well as cell surface localization (small magnification; Supplemental Fig. 4). Judging from the results, we felt that immunofluorescence studies were not sensitive enough to study cell surface localization of each ADAM17 variant in detail. Thus, cell surface detection of ADAM17 was performed in MØP by FACS analysis, as done in HEK cells (Fig. 4F, 4G; compare Fig. 1G). After staining the MØP with an N-terminal ADAM17 Ab and gating for single cells and live cells (7-AAD negative), cells were plotted for secondary Ab signals (488 nm; Fig. 4F). Interestingly, as already indicated by immunofluorescence studies (Supplemental Fig. 4), the ADAM17 Δ700 mutant was found to a lesser extent in cell surface structures compared with all other ADAM17 mutants and ADAM17 wt (Fig. 4G). But judging from the ELISA data, surface localization of the ADAM17 Δ700 was abundant enough for sufficient TNF-α and TNFR_II_ shedding, which was comparable to ADAM17 wt (Fig. 4A, 4B).

To study intracellular ADAM17 regulation and transport in more detail, endogenous protein levels of the transmembrane protein iRhom2, which has been described to be required for ADAM17 ER-to-Golgi transport, cell surface stability, and protease activity (42, 43) were analyzed (Fig. 4H, 4I). Immunoblots of endogenous iRhom2 revealed a similar expression of the protein in M-MØ and GM-MØ, with slightly higher protein levels in GM-MØ (Fig. 4H). Furthermore, immunofluorescence stainings indicated ER as well as cell surface distribution of endogenous iRhom2 in cells of the macrophage progenitor cell system (M-MØ and GM-MØ; Fig. 4I).

Overall, our results suggest that cleavage of the prodomain of ADAM17 at both furin cleavage sites was required for efficient ADAM17 activity in MØP-derived macrophage- and dendritic-like cells, whereas the cytoplasmic tail and its posttranslational modifications seemed to be dispensable for ADAM17 function, but might rather play a role in efficient cell surface trafficking, especially in immune cells.

### Analysis of podosomes, a physiologic readout to validate ADAM17 function in myeloid cells

**FIGURE 5.**
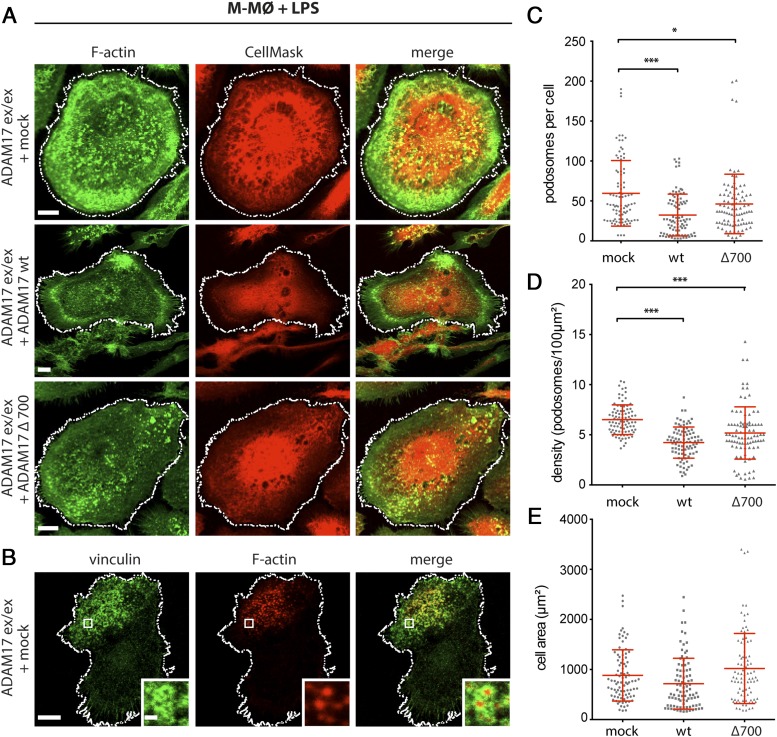
Podosome formation as physiological readout for ADAM17 function. (**A**) Podosomes were visualized by immunofluorescence stainings in LPS-stimulated M-MØ with ADAM17-deficient background (ADAM17^ex/ex^) either expressing empty vector (mock), ADAM17 wt, or cytoplasmic deletion mutant ADAM17 Δ700. Podosomes were visualized by staining F-actin with Alexa Fluor 488–labeled phalloidin (green), and cell membranes were highlighted by using a CellMask staining (red) to clearly distinguish individual cells for quantification. Scale bar, 10 μm. (**B**) As a second marker for podosomes, vinculin, a component of the characteristic podosome ring structure, was stained using a specific primary Ab (green). F-actin staining is shown in red. Scale bar, 10 μm; insets: 1 μm. Quantification of podosome staining in mock, wt, and Δ700-reconstituted cells expressed as podosomes per cell (**C**) and podosome density (podosomes/100 μm^2^) (**D**). (**E**) For analysis of podosome number and density, the cell area of M-MØ was determined by a cell-surrounding white line [as shown in (A) and (B)]. No statistical difference was found between the cell area of mock, ADAM17 wt, and ADAM17 Δ700–reconstituted M-MØ (*n* = 3 × 30; each time, 30 cells were analyzed in three independent cell differentiations). A one-way ANOVA followed by a Tukey multiple comparison test was applied. *p < 0.05, ***p < 0.005.

Podosomes are found in cells derived from the monocyte lineage, including macrophages and dendritic cells (44–47), and have been suggested to be involved in cell migration and invasion (44, 45, 47). Podosomes consist of a core of F-actin, which is typically surrounded by a ring of actin-regulatory proteins like integrins, paxillin, vinculin, and talin (45). The requirement of ADAM17 in TLR-induced disassembly of podosomes was reported (48). In this study, we use podosome number and distribution as a further physiological readout to examine ADAM17 function in myeloid cells. Moreover, the regulatory role of the cytoplasmic tail of ADAM17 was analyzed by studying the effect of the cytoplasmic deletion mutant Δ700 on TLR-induced podosome disassembly (Fig. 5A, 5C, 5D). To this end, ADAM17^ex/ex^ MØP reconstituted with ADAM17 wt, and the Δ700 mutant were differentiated to M-MØ and stimulated with the TLR-ligand LPS. Podosomes were visualized by staining F-actin with fluorescently labeled phalloidin and costained for a second podosome marker (vinculin) to highlight the presence of the podosome ring structure to distinguish them from simple actin dots (Fig. 5A, 5B). A cell mask staining was used to visualize cell membranes (Fig. 5A), to distinguish single cells for quantification, and to calculate cell area, which did not change between mock-transfected and ADAM17 wt or Δ700 variant (Fig. 5E). Both, number as well as density of podosomes per cell were significantly reduced when ADAM17 wt or the Δ700 mutants were reconstituted in comparison with mock-transfected cells (Fig. 5A, 5C, 5D). This emphasizes the significant role of ADAM17 in podosome disassembly and suggests that a truncated ADAM17 C terminus of 6 aa is sufficient for keeping the equilibrium of podosome assembly and disassembly in macrophages (M-MØ). These data identify the MØP system as a well-suited cell model to study podosome regulation, which also seems to be a robust physiological readout assay for ADAM17 function in monocytic cells. The results underline the importance of ADAM17 on podosome disassembly and thus cell motility and normal cell function of macrophages.

## Discussion

Among the ∼80 ADAM17 substrates identified so far (3), the protease has shown to be especially important for cleavage of many regulators involved in immune and inflammatory responses as well as cancer development, including membrane-tethered pro–TNF-α, both TNF-α receptors (TNFR_I_ and TNFR_II_), IL-6R, ligands of the EGF-R (e.g., TGF-α and amphiregulin), and adhesion molecules (e.g., L-selectin and ICAM-1) (3, 6). Because inflammation is characterized by high levels of TNF-α and IL-6, and ADAM17 is essentially involved in the regulation of both cytokine pathways, it makes the metalloprotease a central player in inflammatory processes (6). Moreover, macrophages are the major cell type releasing TNF-α (49), further pointing to ADAM17 as an important regulator of inflammation in this cell type. In addition, the broad substrate repertoire of ADAM17 makes it possible that this protease is involved in pro- as well as anti-inflammatory pathways (6). For instance, TNF-α signaling can be activated by ADAM17-dependent release of the cytokine from the cell surface as well as attenuated by cleavage of TNFR, which act as antagonists of TNF-α (50). The fundamental regulatory function of ADAM17 in governing inflammatory responses requires strict regulation of protease activity. The cellular mechanisms of ADAM17 maturation and activation as well as substrate recognition are not completely understood. Numerous studies addressed the role of posttranslational modifications and their impact on ADAM17 activity. So far, most overexpression studies to analyze the biological behavior of specific ADAM17 mutants have mainly been performed in ADAM17-deficient mEF or HEK cells (13, 15, 24, 26, 27). These nonimmunological cell types have the disadvantage that heterologous substrates and nonphysiologic stimuli need to be employed to evaluate ADAM17 function. Thus, in most experimental setups, cells were cotransfected with ADAM17 substrates such as pro–TNF-α or TGF-α and various ADAM17 mutants, and shedding was induced by PMA (19). The differences of the applied cell systems and the nonphysiological conditions make interpretation as well as comparison of the different studies difficult. The continuing controversy regarding the role of posttranslational events during ADAM17 activation motivated us to establish a more physiological cell model to study ADAM17 maturation in primary cells of the immune system.

We, in this study, introduced an immune cell system on an ADAM17-deficient background (5), which can be genetically modified and thus was used for expression and analysis of ADAM17 mutants. A major advantage of the HoxB8-immortalized macrophage progenitor cell line (31, 32) is that cells can be further differentiated into macrophage- and dendritic-like cells (M-MØ and GM-MØ). Because these cells endogenously express immunologically relevant ADAM17 substrates like TNF-α and TNFR_II_, these substrates were used as a readout for ADAM17 activity. A further benefit of using immune cells is the possibility to work with physiologic stimuli such as the TLR agonists LPS or zymosan.

We, in this study, verified the proteolytic activity of the ADAM17 Δ700 mutant, which contains six residual intracellular amino acids C-terminal of the transmembrane region, because in previous studies, a complete removal of the transmembrane region resulted in a complete inactive protease (26). The role of the cytoplasmic tail in function and regulation of the ADAM17 protease is still controversially discussed in the field. A reason for the divergent results published over the recent years could be the use of various experimental set-ups, including cell systems, choice of analyzed substrates, and the design of the ADAM17 constructs, which often contained charged C-terminal protein tags in the transmembrane region, as indicated by comparison of previous studies (21–27, 51).

In this study, we analyzed the activity of the ADAM17 Δ700 mutant on the cell surface of HEK as well as MØP by using a life cell activity assay. Applying the same experimental readout in both cell lines, ADAM17 Δ700 was found to a lesser extent on the cell membrane as compared with the ADAM17 wt and other ADAM17 variants in the immune cell system (MØP). This could indicate a role of the cytoplasmic tail for trafficking and thus cell surface localization in immune cells compared with nonimmune cells. However, more studies are needed to decipher a possible regulatory impact of the cytoplasmic tail of ADAM17 and to solve the discrepancy in the field about the role of its membrane-proximal portion (21–26, 51).

Shedding activity of the ADAM17 Δ700 mutant questions the role of intracellular phosphorylation in ADAM17 activation (24–26). Nonetheless, phosphorylation of ADAM17 has been regarded as a hallmark of ADAM17 activity (6, 16–18). We analyzed the impact of PLK2-mediated phosphorylation at Ser^794^ on ADAM17 activity because the activity of PLK2 had been predicted to be a regulator of ADAM17 activity (15). Thus, we analyzed the S794A mutant and found no interference with ADAM17 activity in mEF, HEK, and in all MØP-derived cell types. Further, this mutant was found localized at the cell membrane in all analyzed cells.

Activation of ADAM17 by furin-dependent removal of the autoinhibitory propeptide seems to be complex. The proprotein convertase furin removes the prodomain within the Golgi apparatus on the ds cleavage site, which is located between the prodomain and the catalytic domain (11, 12). A recent study identified an additional furin cleavage site within the prodomain (13). In our study, the *RVNG* us mutant was processed by recombinant furin, although levels of the mature (∼90 kDa) form of ADAM17 were less compared with the wt (Fig. 1I). In contrast, the ADAM17 *RVNG* ds only showed a small shift in protein size, which might indicate furin cleavage at the remaining us cleavage site (Fig. 1I, 1J). Both *RVNG* mutations showed impact on ADAM17 activity. In the mEF and HEK cell system, both furin site mutants were found at the cell surface (Fig. 1G), but the *RVNG* us mutant showed less proteolytic activity (Fig. 1H) as also recently described in nonimmunological cell lines (13). In contrast, both *RVNG* mutants showed little proteolytic activity in MØP-derived cells, although located at the cell membrane. Interestingly, it was observed earlier in COS-7 cells that inhibition of proprotein convertases had no effect on ADAM17 shedding activity (24), indicating that the role of furin processing of ADAM17 is not completely understood and will have to be studied in particular in myeloid cells.

Activation of ADAM17 might be cell type–specific, depending on the expression and availability of cofactors influencing ADAM17 biology. Recently, iRhom2 has been described to be such a cofactor (43, 52, 53), showing to be crucially involved in ADAM17 trafficking, stability, and activation (42). Phosphorylation of the cytoplasmic tail of iRhom2 (42) might be the reason for the dispensability of the ADAM17 cytoplasmic portion upon PMA stimulation.

In myeloid cells, ADAM17 is critically involved in podosome disassembly (48). In this study, we showed that the generated MØP-derived macrophage-like cells can be used to study podosome disassembly. In this physiologic cell model, substrates of ADAM17 and the disassembly mechanism involved in podosome loss can be studied, which will be important for the functional understanding of these actin-rich adhesion structures that are crucial for cell migration and matrix invasiveness of macrophages and dendritic cells (54).

## Supplementary Material

Data Supplement
