# Supplementary material for: Structural and Functional Analyses of the Shedding Protease ADAM17 in HoxB8-Immortalized Macrophages and Dendritic-like Cells
Source: J Immunol. 2018 Oct 24;201(10):3106–18. doi: 10.4049/jimmunol.1701556 (PMC6215251; doi:10.4049/jimmunol.1701556)
Supplement: Data Supplement [file JI_1701556.zip › JI_1701556_Supplemental_Figures_1.pdf]

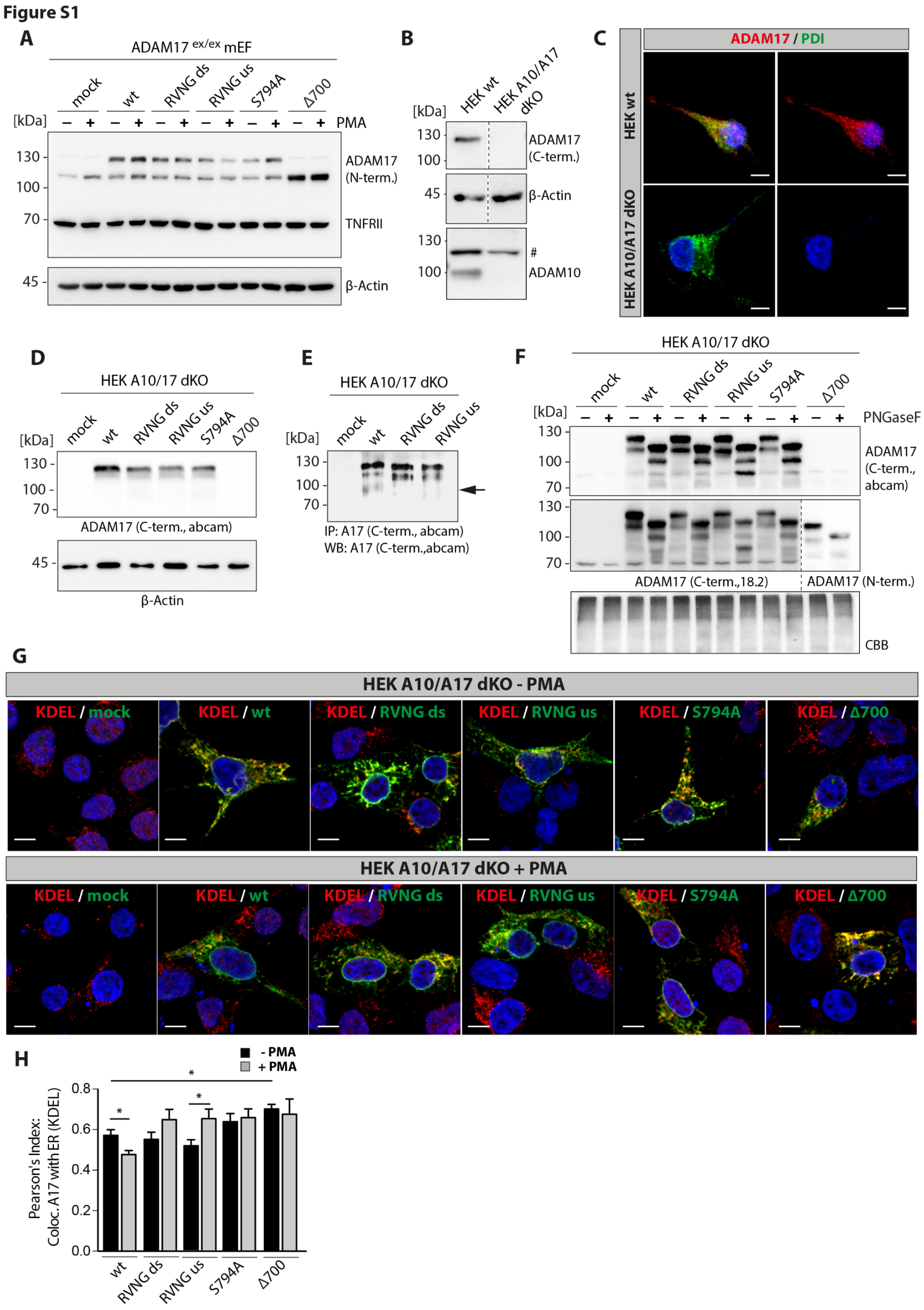

Figure S1: Characterization of ADAM17 mutants in mEF and ADAM10/17 dKO HEK cells. Corresponding to Figure 1. **A)** Representative immunoblot of endogenous TNFR$_{II}$ protein level in ADAM17$^{ex/ex}$ mEF after transfection with respective ADAM17 mutant under unstimulated and 2-h PMA-stimulated conditions. For detection of ADAM17 an N-terminal antibody was utilized (10.1). ß-Actin was used as loading control. **B)** Double-deficient ADAM10 and ADAM17 (A10/A17 dKO) HEK cells were derived by CRISPR/Cas9. Representative immunoblot stained for ADAM17 (specific anti-human ADAM17, A300D, (55)) and ADAM10 verifying deficiency of both proteases. ß-Actin was used as loading control. # indicates unspecific antibody band. **C)** ADAM17-deficiency was further characterized by immunofluorescence analysis, utilizing a human ADAM17-specific antibody (A300E, (55)) and PDI as ER-marker. Absence of red signal in HEK A10/A17 dKO in comparison to wildtype (wt) HEK cells indicates knock-out or protein. Scale bar: 10µm. **D)** Immunoblot of overexpressed ADAM17 in lysates of ADAM10/17-deficient HEK cells utilizing a commercial C-terminal antibody (abcam). As loading control ß-Actin was used. **E)** Western blot of precipitated ADAM17 from ADAM10/17-deficent HEK cells after reconstitution with ADAM17 wt as well as RVNG ds and RVNG us mutants. Top, precipitation of ADAM17 by c-terminal ADAM17-specific antibody (abcam). Arrow indicates mature form of ADAM17 with an approximate size of 90 kDa. **F)** Immunoblot of ADAM17 in HEK A10/17 dKO cells reconstituted with respective ADAM17 mutants after treatment with PNGaseF utilizing three different antibodies: C-terminal (abcam; 18.2) and for detection of Δ700 mutant: N-terminal (10.1). **G)** Immunofluorescence study in HEK A10/A17dKO cells overexpressing ADAM17 variants, indicating ADAM17 in green and KDEL as ER-marker in red. The upper panel shows cellular ADAM17 localization without stimulation (-PMA) and the lower panel after stimulation with PMA. Scale bar: 10µm. **H)** Colocalization of each ADAM17 variant with the ER (KDEL) was calculated and is expressed as Pearson's index. Values can range between 0 and 1, with value 1 representing a 100% colocalization. n=3 (at least 5 transfected cells were analyzed from 3 independent stainings).

Figure S2

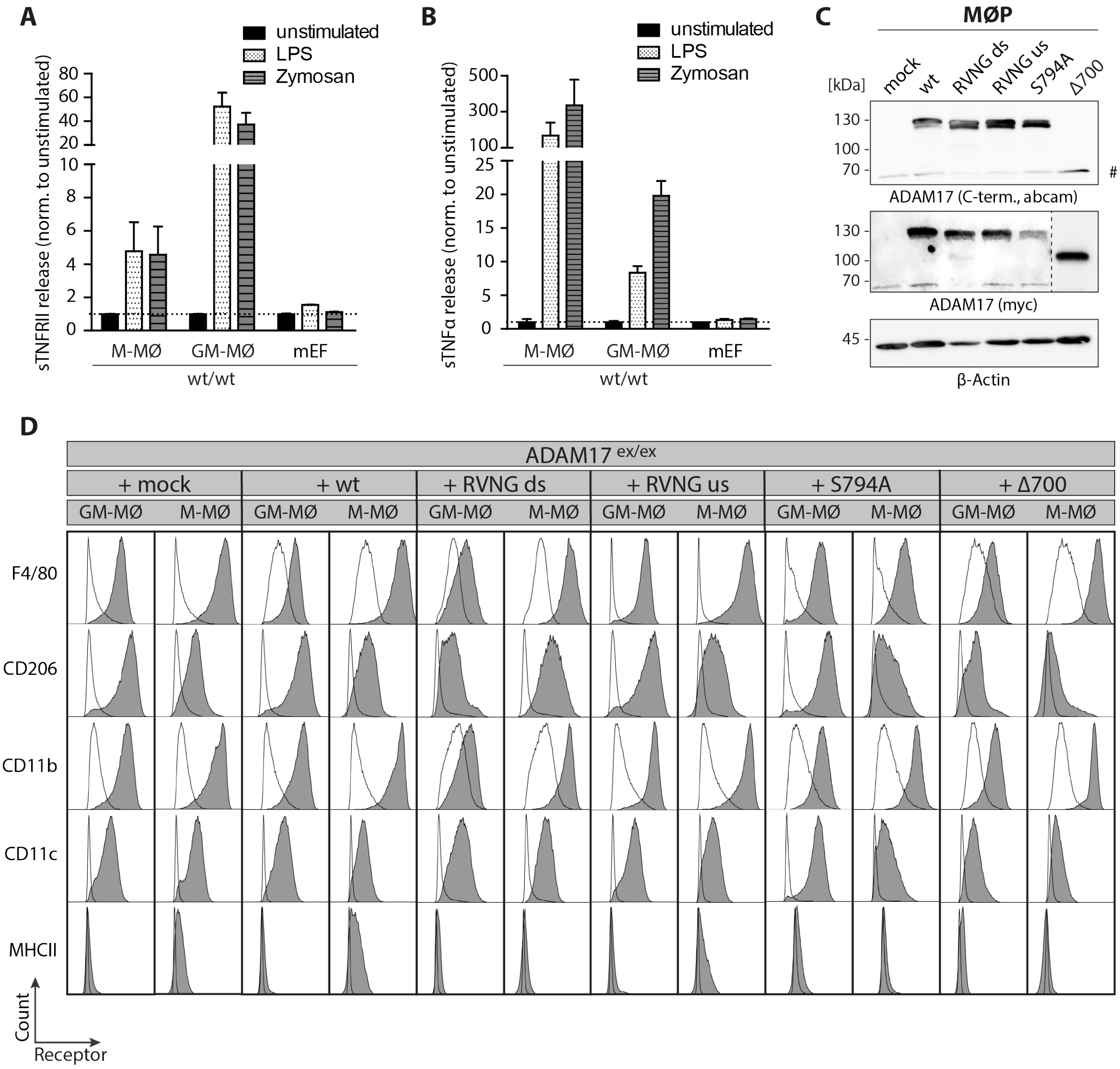

Figure S2: Characterization of the MØP cell system: function and expression of ADAM17. Corresponding to Figure 1-3. **A-B)** Comparison of ADAM17 activation and shedding activity between mEF and M-MØ / GM-MØ cells: ELISA assays of sTNFR$_{II}$ **(A)** and sTNFα **(B)** measured from cell supernatant of M-MØs and GM-MØs as well as mEF, all derived from wildtype (wt/wt) animals. Cells were stimulated with LPS (white, black dots) or zymosan (dark grey), results are normalized to untreated (unstimulated) cells. n=3-9. **C)** Immunoblot of ADAM17 variants in stably transduced MØP cells. In addition to C- and N-terminal ADAM17 antibodies (18.2; 10.1; see Figure 3A), a commercially available C-terminal antibody (abcam) as well as anti-myc antibody were used to detect C-terminal myc-tagged ADAM17 variants. ß-Actin was used as loading control. # indicates unspecific antibody band. **D)** FACS analysis of MØP cells in ADAM17$^{ex/ex}$ background were reconstituted with respective ADAM17 variant (wt, *RVNG* ds, *RVNG* us, S794A and Δ700), differentiated to M-MØ and GM-MØ and analyzed for immune cell surface markers. Shaded histograms represent the receptor staining of each respective differentiated cell (M-MØ/GM-MØ) and bold lines indicate receptor signal of undifferentiated MØP cell. Signal of the receptor staining increases according to differentiation and seems to be independent of ADAM17 variant. The histograms are representative of plots from three independent FACS experiments for each marker.

Figure S3

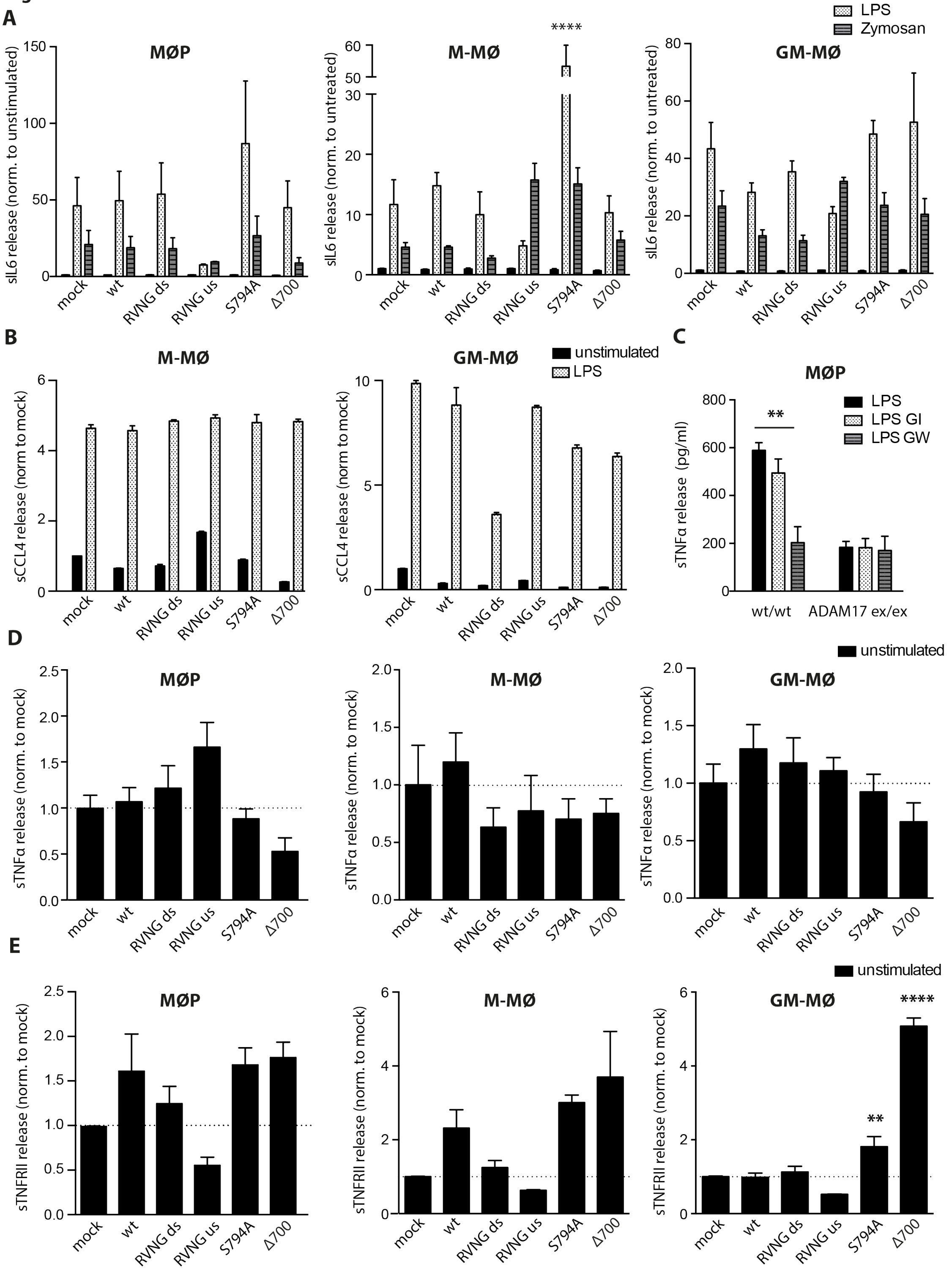

Figure S3: Analysis of ADAM17 function in MØP, M-MØ and GM-MØ cells by ELISA. Corresponding to Figure 4. **A)** IL6 shedding ELISA assay of cell supernatant of ADAM17-reconstituted MØP, M-MØ and GM-MØ cells without stimulation (untreated, black) and after stimulation with LPS (light grey) and zymosan (dark grey). Results are shown normalized to each respective untreated sample. n=9 derived from 3 independent rounds of differentiation. **B)** ELISA assay of soluble chemokine (C-C motif) ligand 4 (sCCL4) level in supernatants of ADAM17-reconstituted M-MØ and GM-MØ cells without stimulation (untreated, black) and after stimulation with LPS (light grey). Results are shown normalized to mock transfection. n=3 from three independent rounds of differentiations. **C)** TNF*α* ELISA assay of cell supernatant of wt and ADAM17$^{ex/ex}$ MØP cells after stimulation with LPS. Furthermore, the ADAM10 inhibitor (GI, light grey) and ADAM10 and ADAM17 inhibitor (GW, dark grey) were applied to validate specificity of utilized ELISA for ADAM17 activity. n=3. TNF*α* **(D)** and TNFR$_{II}$ **(E)** shedding ELISA assay of cell supernatant of unstimulated (untreated) ADAM17-reconstituted MØP, M-MØ and GM-MØ cells. Results were normalized to the mock transfected control and dotted lines indicate baseline shedding activity. n=9 derived from 3 independent rounds of differentiation. Statistical significances are shown in comparison to ADAM17 wt, utilizing a one-way ANOVA, followed by a Tukey multiple comparison test. **p<0.01, ****p<0.001.

Figure S4

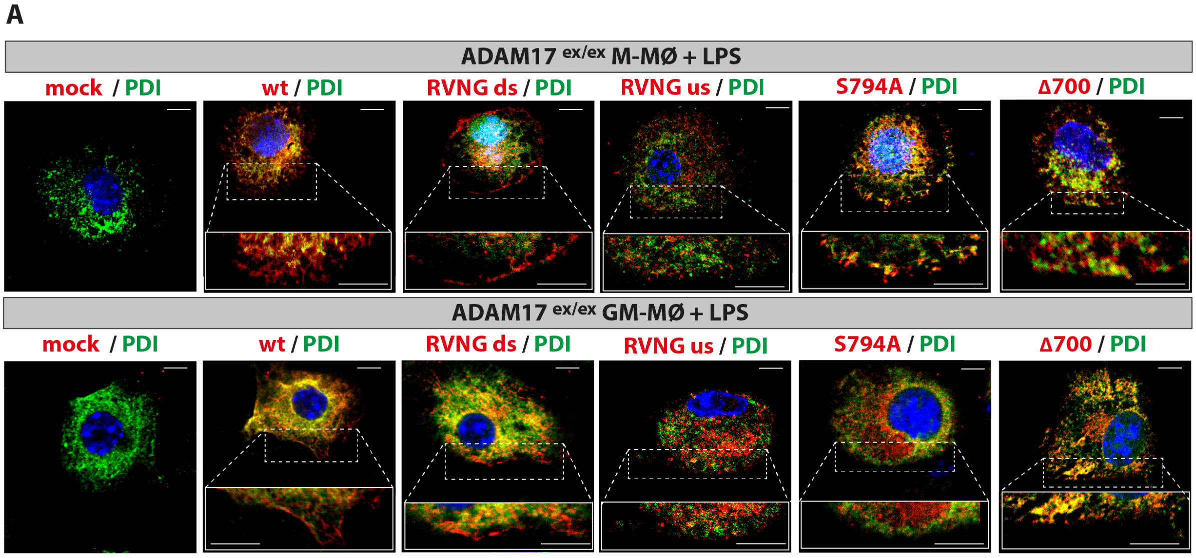

Figure S4: Immunofluorescence analysis of cellular localization of ADAM17 variants in MØP-derived cells. Corresponding to Figure 4. **A)** Immunostaining of M-MØ and GM-MØ cells stably reconstituted with respective ADAM17 variant. A costaining was performed by utilizing an anti-myc antibody for ADAM17 (red) and PDI to visualize ER structures (green). The white box shows magnification of cell surface structures. Scale bar: 10µm. Pictures show representative cells, which were observed in at least three independent differentiations.
